# Impact of a molecular syndromic panel on *Clostridioides difficile* detection and clinical interpretation

**DOI:** 10.1017/ice.2025.10313

**Published:** 2026-01

**Authors:** Nancy Matic, Shayan Shakeraneh, Jennifer Bilawka, Leah Gowland, Willson Jang, Colin Lee, Victor Leung, Michael Payne, Aleksandra Stefanovic, Christopher F. Lowe, Marc G. Romney

**Affiliations:** 1 Department of Pathology and Laboratory Medicine, University British Columbia, Vancouver, Canada; 2 Division of Medical Microbiology and Virology, St. Paul’s Hospital, Vancouver, Canada; 3 Infection Prevention and Control, Providence Health Care, Vancouver, BC, Canada; 4 Antimicrobial Stewardship Program, Providence Health Care, Vancouver, Canada

## Abstract

After implementation of a molecular syndromic panel for infectious diarrhea, a significantly greater proportion of *C. difficile* results were classified as colonization rather than infection compared to the pre-implementation period. Routine *C. difficile* reporting from multiplex panels should be re-evaluated to minimize diagnostic uncertainty in some patients.

## Introduction

Diagnosis of *Clostridioides difficile* infection (CDI) is challenging, with multiple testing algorithms proposed^1,2^. With increased availability of molecular syndromic panels in clinical laboratories, many patients without typical risk factors are routinely tested for *C. difficile* using multiplex assays, adding further complexity to interpretation of results. Some groups have suggested suppressing *C. difficile* results from panels^3^; performing additional confirmatory testing with enzyme immunoassays (EIAs) or secondary molecular tests^4,5^; or, sending panel results for clinical review. Furthermore, the impact of panels on *C. difficile* positivity rates and clinical outcomes has not been fully characterized.

Our laboratory implemented an infectious diarrhea panel (IDP) in late 2023. This multiplex panel is performed on all stool samples submitted from outpatients and inpatients (<72 hours of admission) presenting with acute gastroenteritis. To assess the impact of IDP on CDI rates, we investigated *C. difficile* laboratory results and clinical interpretation for a 6-month period pre- and post-IDP implementation.

## Methods

Prior to IDP implementation, patients presenting to two acute tertiary care hospitals (St. Paul’s Hospital and Mount Saint Joseph Hospital, Vancouver, BC) and nearby long-term care sites and outpatient clinics with clinical suspicion for CDI had stool samples submitted for dedicated two-step C*. difficile* testing. Stool samples first underwent molecular detection of the *tcdB* gene (Xpert® *C. difficile*/Epi, Cepheid). Positive samples underwent further analysis by enzyme immunoassay (EIA) for direct detection of *C. difficile* toxin A/B and glutamate dehydrogenase (GDH) antigen (C. Diff QuikChek Complete, Techlab). If toxin was not detected by EIA, final results were reported as “Indeterminate” rather than “Positive.” Clinical review of each inpatient case (including patients admitted from the Emergency Department [ED]) was conducted by the hospital’s antimicrobial stewardship program (ASP), determining whether patients were infected (new-onset ≥3 loose stools in 24 hours without an alternate diagnosis) or colonized (alternate diagnosis identified based on clinical review of stool charts, medications [e.g., laxatives or other medications associated with diarrhea], laboratory results, underlying conditions, and final discussion with the patient’s attending physician)^6,7^.

After implementation of IDP, dedicated two-step C*. difficile* testing remained available for inpatients when clinically suspected; however, all patients presenting with acute gastroenteritis for any reason had stool samples tested by IDP (BioFire® FilmArray® Gastrointestinal [GI] Panel, bioMérieux), replacing traditional stool bacterial culture and ova & parasite examination. Samples with *C. difficile* incidentally detected by IDP underwent further testing by EIA. Clinical review by infection prevention and control (IPAC) and ASP was conducted for all inpatient cases, as previously described^6,7^.

Results were retrospectively reviewed for a 6-month period after implementation of IDP (February-July 2024). Rates of positivity and colonization versus CDI were compared to the same 6-month period before IDP implementation (February–July 2023). Fisher’s exact test (GraphPad QuickCalcs) was used for statistical analysis where applicable, with *p* < 0.05 considered significant.

## Results

In the post-IDP period, *C. difficile* was the most frequently detected pathogen on IDP in our patient population (10.8% detection rate). A higher number of stool samples underwent *C. difficile* testing compared to the pre-IDP period (1,661 vs 1,049), with a greater proportion submitted from ED and outpatients with a younger median age (Table 1). Of note, the number of orders for dedicated *C. difficile* testing decreased by nearly half compared to the pre-IDP period. Of samples testing positive for *C. difficile* by IDP, a significantly higher proportion (27%) tested negative for both GDH and toxin EIA compared to dedicated *C. difficile* testing in the same post-IDP period (6%, *p* < 0.001) and the pre-IDP period (11%, *p* < 0.001).

**Table 1.** Results of stool samples submitted to the microbiology laboratory during the pre-IDP (February to July 2023) and post-IDP (February to July 2024) periods

	Pre-IDP Dedicated *C. difficile* order (Xpert *C. difficile*/Epi)	Post-IDP Infectious Diarrhea Panel (BioFire GI Panel)	Post-IDP Dedicated *C. difficile* order (Xpert *C. difficile*/Epi)
Total stool samples submitted	1081	1257	560
Ordering Location			
Outpatient Clinic	109 (10%)	243 (19%)	25 (5%)
Emergency Department	418 (39%)	717 (57%)	40 (7%)
Inpatient Ward	554 (51%)	297 (24%)	495 (88%)
Median Age (years, range)	62 (4–100)	52 (0–98)	64 (19–96)
Stool samples rejected by lab^1^	32 (3%)	137 (11%)	19 (3%)
No. of stool samples tested	1049	1120	541
**Results**			
*C. difficile* detected by NAT	134 (13%)	121 (11%)	84 (16%)
EIA (QuikChek) results			
GDH Neg, Toxin Neg	14 (11%)*	33 (27%)	5 (6%)*
GDH Pos, Toxin Neg	70 (52%)	55 (46%)	44 (52%)
GDH Pos, Toxin Pos	50 (37%)	32 (26%)	35 (42%)
GDH Neg, Toxin Pos	0	1 (1%)	0
Final Report			
Positive	50 (5%)	33 (3%)	35 (7%)*
Indeterminate	84 (8%)	88 (8%)	49 (9%)

* *p* < 0.001 compared to Infectious Diarrhea Panel (IDP).

NAT, nucleic acid amplification test, either by BioFire GI Panel or Xpert *C. difficile*/Epi test as indicated.

EIA, enzyme immunoassay.

GDH, glutamate dehydrogenase antigen.

^1^ Stool samples rejected if <6 on Bristol Stool Chart or repeated within 7 days. IDP orders were additionally rejected if sample collected >72 hours after hospital admission.

Clinical review of inpatient cases revealed a significantly greater proportion of patients tested by IDP was interpreted as “Colonized” compared to patients in the pre-IDP period (46.9% vs 27.5%, *p* = 0.01). Even when comparing to patients who had dedicated *C. difficile* tests performed within the same post-IDP period, colonization rates were higher among patients tested by IDP only, although this difference did not quite reach statistical significance (46.9% vs 37.3%, *p* = 0.30). Clinical outcomes including critical care admission, surgical intervention, and 30-day all-cause mortality did not significantly differ between the pre- and post-IDP periods (Table 2).

**Table 2.** Classification of inpatient *C. difficile* cases by clinical review, including clinical outcomes in the pre- and post-IDP periods

	Number of cases	Hospital-onset	ICU admission	Surgical procedure	All-cause mortality
**Pre-IDP**					
Colonization	28 (27.5%)	N/A	2 (7.1%)	0	1 (3.6%)
Infection	74 (72.5%)	34 (45.9%)	4 (5.4%)	2 (2.7%)	6 (8.1%)
**Post-IDP (patients with only IDP ordered)**					
Colonization	30 (46.9%)*	N/A	0	1 (3.3%)	0
Infection	34 (53.1%)	10 (29.4%)	0	2 (5.9%)	1 (2.9%)
**Post-IDP (patients with dedicated** ***C. difficile*** **test ordered)**					
Colonization	28 (37.3%)	N/A	3 (10.7%)	2 (7.1%)	3 (10.7%)
Infection	47 (62.7%)	33 (70.2%)**	1 (2.1%)	0	7 (14.9%)

* *p* = 0.01 compared to the pre-IDP (Infectious Diarrhea Panel) period.

**p < 0.01 compared to the pre-IDP period and to IDP results in the post-IDP period.

ICU, intensive care unit.

## Discussion

Molecular syndromic panels have several advantages including improved efficiency and turnaround time; however, routine testing for *C. difficile* regardless of patients’ pre-test probability (or prevalence of the condition in the population being tested) may not be optimal. Patients with CDI typically have risk factors and clinical presentations that differ from those with foodborne or community-acquired infectious diarrhea, and molecular assays for *C. difficile* toxin genes may be detecting asymptomatic carriers of *C. difficile* rather than those with CDI^8^. Our study demonstrates two different patient populations being tested for *C. difficile* in the pre- and post-IDP periods, with an impact on clinical interpretation of the results.

The detection rate of *C. difficile* by IDP in our study was similar to what has been previously described in other centres using the BioFire GI panel (9.7–16.3%)^3–5^. The majority of these samples tested negative for toxin EIA in our study, consistent with previous reports (57–78%)^4,9,10^. Our laboratory previously observed negative EIA toxin in 60% of samples when using a laboratory-developed test^7^ and 67% when using Xpert^6^ as the initial molecular assay in a two-step algorithm, which increased to 73% using IDP in this study. A key finding was the significant increase in the proportion of samples testing negative for both GDH and toxin EIA compared to our pre-IDP period. No samples with both negative GDH and toxin EIA were observed in our centre’s previous study using a laboratory-developed assay as the initial molecular test^7^.

Clinical review was conducted on only a subset of the cases (inpatients), which demonstrated a higher proportion being interpreted as “Colonization” when tested by IDP. This increase may be driven by the higher rate of GDH and toxin EIA negative samples in this cohort, and also potentially reflects the lower CDI pre-test probability in patients undergoing IDP. The significant decrease in dedicated *C. difficile* orders during the post-IDP period suggests many clinicians ordered IDP instead of dedicated *C. difficile* testing. This may be concerning from an ASP perspective, as previous studies have demonstrated patients with positive *C. difficile* results by a molecular assay are likely to receive treatment regardless of their pre-test probability or EIA results^10^. Unfortunately, IPAC and ASP surveillance teams in our centre are not able to review *C. difficile* results from outpatients and those discharged from ED; it is unclear how clinicians in the community may be interpreting and managing indeterminate *C. difficile* IDP results.

This study has additional limitations, including potential confounding factors during the pre- and post-IDP periods that may have affected *C. difficile* positivity rates and clinical interpretation; however, this study design was necessary to evaluate real-world data after implementation of a new testing method. Additional outcomes of interest including antibiotic usage and symptom resolution are not routinely collected by IPAC and ASP teams and were not available for analysis. Sample size was limited due to *C. difficile* positivity rates in our patient population.

With a two-step algorithm in use, the IDP did not significantly alter *C. difficile* “Positive” and “Indeterminate” rates, although a greater proportion of inpatient cases was interpreted as colonization after clinical review. To prevent potential over-treatment of *C. difficile* IDP results, it would be important to continue the two-step algorithm and clinical review, and consider suppression of *C. difficile* results from molecular syndromic panels in populations where clinical consultation is not available or for which colonization rates are high.
